# Molecular Modeling of Weakly Caking Coal and the CO_2_ Inhibition Mechanism of Coal–Oxygen Complexation

**DOI:** 10.3390/molecules31122108

**Published:** 2026-06-15

**Authors:** Xiaoyue Zhao, Xihua Zhou, Wenqing Wang

**Affiliations:** 1 College of Safety Science & Engineering, Liaoning Technical University, Huludao 125105, China; 2 Key Laboratory Mine Thermodynam Disasters & Control, Ministry of Education, Liaoning Technical University, Huludao 125105, China

**Keywords:** weakly caking coal, molecular structure, Grand Canonical Monte Carlo, O_2_-CO_2_ competitive adsorption, adsorption heat

## Abstract

To elucidate the molecular structural characteristics of weakly caking coal and the microscopic mechanism by which CO_2_ inhibits coal–oxygen complexation, a weakly caking coal sample from the Dahaize coal mine in Shaanxi, China, was investigated using proximate and ultimate analyses, FTIR, XPS, and ^13^C NMR. On this basis, a representative coal macromolecular model was constructed and further analyzed using density functional theory (DFT) and grand canonical Monte Carlo (GCMC) simulations. The molecular formula of the representative weakly caking coal from the Dahaize mine (RNM) unit was determined as C_176_H_156_N_2_O_19_S_2_. The aromatic carbon fraction was 65.41%, and the bridge carbon/peripheral carbon ratio was 0.25, indicating a certain degree of aromatic condensation but a limited content of highly fused aromatic structures. DFT calculations revealed that the reactive sites were mainly located around edge oxygen-containing functional groups and bridging structures, with a maximum Fukui index of approximately 0.024. Adsorption simulations showed that O_2_ and CO_2_ adsorption on RNM followed Langmuir-type behavior over 303.15–363.15 K: adsorption capacity increased with pressure and decreased with temperature. At 8000 kPa, the CO_2_ uptake was approximately 1.6 times that of O_2_. In the binary O_2_-CO_2_ system, CO_2_ preferentially occupied pore surfaces and high-energy adsorption sites, reducing the local enrichment of O_2_. These results provide a molecular-level explanation for the inhibition of coal–oxygen complexation by CO_2_ through competitive adsorption, site shielding, and decreased oxidation probability at active sites.

## 1. Introduction

Weakly caking coal is a low-rank coal with a relatively high content of oxygen-containing functional groups and aliphatic side chains, which makes it prone to low-temperature oxidation and spontaneous combustion during mining, storage, and utilization. Coal–oxygen complexation is generally regarded as the initial stage of coal oxidation, in which oxygen molecules are adsorbed onto active sites of the coal matrix and subsequently participate in radical reactions and functional group transformations. Therefore, clarifying the molecular structure of weakly caking coal and identifying the preferential adsorption and reaction sites of O_2_ are essential for understanding its oxidation activity and for developing effective oxygen-inhibition strategies.

The molecular structure of coal is highly heterogeneous and is composed of aromatic clusters, aliphatic chains, heteroatom-containing functional groups, and cross-linked bridge structures. Conventional experimental techniques, including elemental analysis, FTIR, XPS, and ^13^C NMR, have been widely used to characterize the elemental composition, functional group distribution, heteroatom occurrence, and carbon skeleton of coal. These techniques provide important structural constraints for constructing macromolecular coal models. Previous studies have demonstrated that combining multi-scale experimental characterization with molecular simulation is an effective approach for revealing the structural evolution and reaction behavior of coal at the molecular level. However, most existing studies have focused mainly on coal structural characterization or thermal reaction behavior, whereas the relationship between molecular active sites, O_2_ adsorption, and CO_2_ competitive inhibition remains insufficiently clarified.

Since the 1980s, extensive work has been devoted to average structural model construction, three-dimensional reconstruction, and molecular dynamic simulation of coal. Representative early studies include the structural bases for coal pyrolysis proposed by Solomon and co-workers [1] and the statistical structural model concept developed by Given [2]. In the 1990s, Wiser established a more systematic framework for average structural models based on elemental analysis, solid-state ^13^C NMR, and infrared data [3]. Faulon introduced computer-assisted stochastic assembly to generate three-dimensional coal models constrained by experimental parameters, which is widely regarded as a key milestone in digital coal modeling [4]. More recently, Mathews et al. developed a refined three-dimensional model for Illinois No. 6 bituminous coal and verified its density and pore-related parameters via molecular dynamics, highlighting a shift from two-dimensional average structures to three-dimensional statistical representations [5]. Ungerer and co-workers further applied molecular simulation to coal–CO_2_/CH_4_ interactions, promoting the use of coal molecular models in Coalbed Methane (CBM) and CO_2_ geological storage. In China, researchers such as Huang XH and Zhang SY constructed average macromolecular models for coals of different ranks using combined ^13^C NMR, XPS, and FTIR constraints [6].

In recent years, considerable attention has been devoted to the inhibitory effect of CO_2_ on coal low-temperature oxidation and spontaneous combustion. In experimental studies, programmed heating, thermogravimetry–differential scanning calorimetry, microcalorimetry, and in situ Fourier transform infrared spectroscopy have been widely employed to evaluate the influence of CO_2_ on coal oxidation behavior. Ding et al. [7] combined experimental analysis with molecular simulation and demonstrated that CO_2_ injection increased the initial CO generation temperature, reduced CO production, and weakened O_2_ adsorption through diffusion displacement and competitive occupation of adsorption sites. Zhou et al. [8] further revealed that CO_2_ affected the evolution of active functional groups and macroscopic gas release during coal low-temperature oxidation. In addition, Haifei Y et al. [9] reported that liquid CO_2_ exhibited both cooling and inerting effects, thereby reducing oxidation heat release and improving the resistance of coal to spontaneous combustion. At the molecular scale, grand canonical Monte Carlo, molecular dynamics, and density functional theory methods have increasingly been applied to elucidate the microscopic mechanism by which CO_2_ inhibits coal–oxygen reactions. Cheng et al. [10] confirmed through combined experimental and simulation approaches that CO_2_ could hinder O_2_ adsorption during the low-temperature oxidation stage of low-rank coal. Wang [11] et al. analyzed competitive adsorption behavior, adsorption capacity, adsorption selectivity, and diffusion coefficients, and found that CO_2_ exhibited a stronger competitive adsorption advantage than O_2_, with this advantage being temperature-dependent. Jia et al. [12] reported that CO_2_ tended to aggregate in coal pores, whereas O_2_ was more discretely distributed; moreover, the adsorption capacity, isosteric heat of adsorption, and adsorption selectivity of CO_2_ were higher than those of O_2_ under identical conditions. Dong et al. [13] also showed that CO_2_ more readily approached adsorption saturation in multicomponent CO/CO_2_/O_2_ systems, indicating its preferential adsorption behavior. From an engineering perspective, CO_2_ injection into goafs has become an important strategy for coal mine fire prevention and control. Si et al. [14]. investigated the safety of CO_2_-based fire prevention technology in goaf-side entry-retaining systems, while Cai et al. [15] proposed coordinated control parameters for gas drainage and CO_2_ inerting in highly gassy and spontaneous-combustion-prone mines. Junhong S [14] further used numerical simulation to examine the effects of CO_2_ injection position and operating parameters on the oxidation zone in goafs. These studies indicate that CO_2_ injection can reduce O_2_ concentration, narrow the oxidation zone, and enhance inerting efficiency. However, its inhibitory performance is strongly affected by injection location, flow rate, air leakage conditions, and goaf structure.

CO_2_ injection is considered a potential method for suppressing coal oxidation because CO_2_ can dilute oxygen, occupy pore spaces, and competitively adsorb on coal surfaces. Nevertheless, the microscopic mechanism by which CO_2_ inhibits coal–oxygen complexation is still not fully understood. In particular, it remains necessary to determine whether CO_2_ suppresses oxidation mainly through physical competitive adsorption, preferential occupation of high-energy adsorption sites, or restriction of O_2_ access to chemically active sites. Since the adsorption behavior of O_2_ and CO_2_ is strongly affected by coal molecular polarity, functional groups, pore structure, and temperature, a combined quantum chemical and molecular simulation approach is required to reveal the interaction mechanism between coal, O_2_, and CO_2_.

In this study, weakly caking coal from the Dahaize coal mine (RNM) in Yulin, Shaanxi Province, China, was selected as the research object. Elemental analysis, FTIR, XPS, and ^13^C NMR were first employed to determine the structural parameters of the coal sample, including aromaticity, aliphatic chain characteristics, heteroatom occurrence, and carbon skeleton distribution. Based on these experimental constraints, a representative RNM macromolecular model was constructed and validated by comparing the simulated and experimental ^13^C NMR spectra. Subsequently, density functional theory calculations were performed to analyze the electrostatic potential distribution, frontier molecular orbitals, and Fukui function-based attack indices, thereby identifying the preferential reactive sites involved in coal–oxygen complexation. Furthermore, grand canonical Monte Carlo simulations were conducted to investigate the adsorption differences between O_2_ and CO_2_ and their competitive adsorption behavior at different temperatures. The objective of this work is to establish a molecular-level correlation among coal structural characteristics, active site distribution, and O_2_-CO_2_ competitive adsorption. The innovation of this study lies in coupling experimentally constrained coal molecular modeling with density functional theory (DFT) reactivity analysis and grand canonical Monte Carlo (GCMC) adsorption simulation, which enables simultaneous identification of oxidation-prone sites and CO_2_ preferential adsorption behavior. The results are expected to provide a theoretical basis for understanding CO_2_ inhibition of coal–oxygen complexation, gas migration and site occupation in coal pores, and the microscopic mechanism of CO_2_-assisted prevention of low-temperature oxidation in weakly caking coal.

## 2. Results

### 2.1. Basic Coal Quality Analysis

A weakly caking coal sample collected from the Dahaize coal mine in Yulin, Shaanxi Province, China, was selected as the research object in this study. Proximate and ultimate analyses were performed to provide elemental constraints for molecular formula determination. C, H, N, and S were measured using an Elementar Unicube organic elemental analyzer, while O was obtained by calculation. Vitrinite reflectance was measured according to GB/T 6948–2008 [16]. Proximate analysis was conducted following GB/T 212–2008 [17]. The elemental and proximate analysis results of the coal samples are summarized in Table 1. In Table 1, M_ad_ represents the moisture content of the coal sample on an air-dried basis, A_ad_ represents the ash content on an air-dried basis, V_ad_ represents the volatile matter on an air-dried basis, and FC_ad_ represents the fixed carbon on an air-dried basis [16].

### 2.2. FTIR-Based Analysis of Functional Groups

#### 2.2.1. Peak Fitting Analysis of FTIR

**Figure 1:**
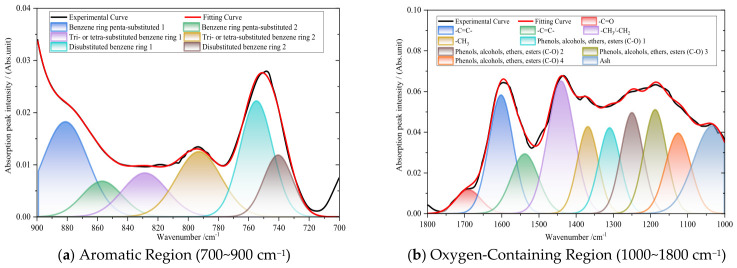

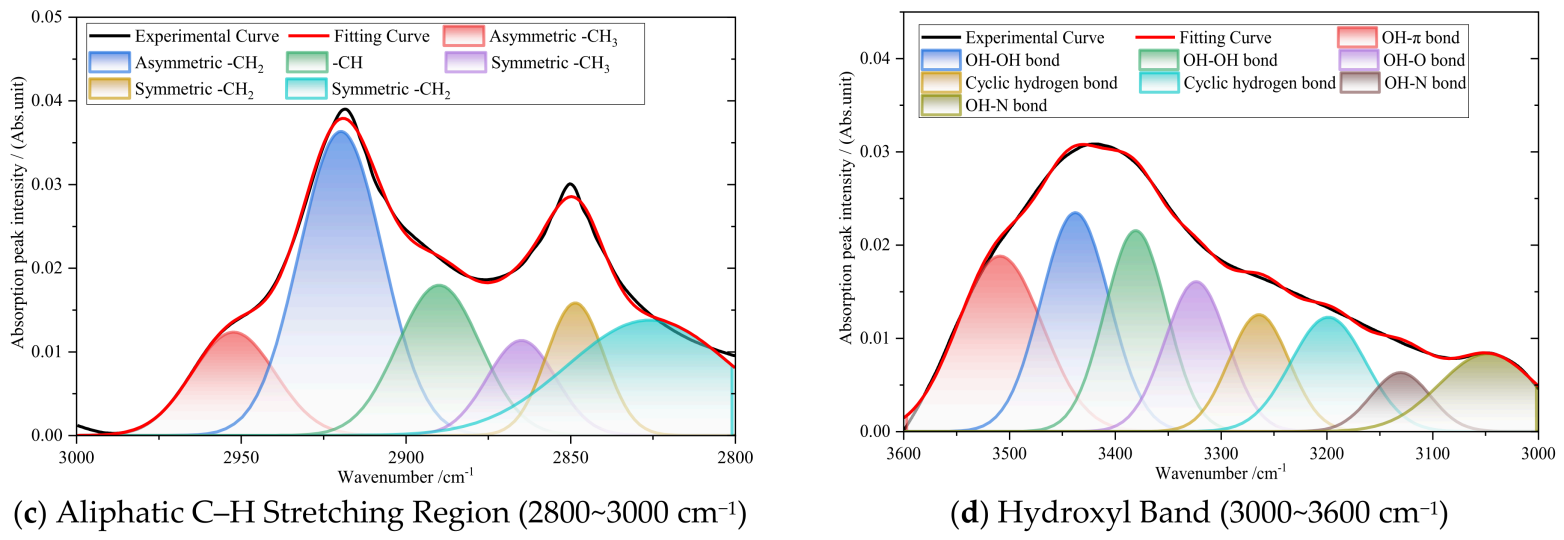
Fitted infrared spectra of the four spectral regions of RNM.

FTIR spectroscopy was used to identify the functional groups and structural parameters of RNM. The spectrum was divided into four characteristic regions: the hydroxyl absorption region (3600–3000 cm^−1^), aliphatic C-H stretching region (3000–2800 cm^−1^), oxygen-containing functional group region (1800–1000 cm^−1^), and aromatic C-H out-of-plane bending region (900–700 cm^−1^). Peak fitting was conducted to quantify the relative distribution of aromatic substitution modes, hydrogen-bonding types, oxygen-containing structures, and aliphatic side chains. The infrared spectral fitting curves for the four regional bands of RNM are shown in Figure 1.

The deconvolution results showed that the peak area assigned to pentasubstituted benzene rings in RNM was 1.03, accounting for 38.5% of the total aromatic substitution region. The peak areas of tri-/tetrasubstituted and disubstituted benzene rings were 0.77 and 0.87, corresponding to 28.8% and 32.5%, respectively. These results indicate that pentasubstituted aromatic units were the dominant benzene ring substitution form in the RNM macromolecular structure, whereas tri-/tetrasubstituted and disubstituted aromatic rings occurred as secondary structural units.

For the hydroxyl region, OH–OH hydrogen bonds exhibited the highest relative proportion, reaching 35.46%, and therefore represented the primary hydrogen-bonding association type. OH–π hydrogen bonds accounted for 19.16%, suggesting strong interactions between hydroxyl groups and aromatic π–electron systems. In addition, cyclic hydrogen bonds contributed 24.84%, indicating the presence of abundant stable associated structures in the coal matrix. These results suggest that hydroxyl self-association and cyclic hydrogen bonding were the dominant association modes in RNM, resulting in a relatively complex hydrogen-bonding network.

In the oxygen-containing functional group region, the total peak-area contribution of C–O stretching vibrations reached 51.07%, indicating that oxygen-containing side chains and ether-bridge structures were abundant in the coal matrix. The combined contribution of aliphatic CH_3_ and CH_2_ vibrations was 25.03%, demonstrating the development of aliphatic chain-substituted structures. The aromatic C=C skeletal vibration bands accounted for 21.06%, suggesting that the coal sample possessed a certain degree of aromatization, although the degree of aromatic condensation remained limited.

In the aliphatic C–H stretching region, the peak-area proportion of CH_2_ groups was 64.04%, markedly higher than that of the CH_3_ groups, which was 19.71%. The calculated A(CH_2_)/A(CH_3_) ratio was approximately 3.26, indicating that methylene segments dominated the aliphatic structures and that the aliphatic side chains were relatively long with a low degree of branching. Moreover, aliphatic C–H vibrations accounted for 16.22%, further confirming the enrichment of aliphatic structures in RNM. Overall, the FTIR fitting results demonstrate that RNM was characterized by abundant oxygen-containing functional groups and aliphatic side chains, together with a developed but weakly condensed aromatic skeleton.

#### 2.2.2. FTIR-Derived Structural Parameters

Aromaticity, aromatic condensation degree, and aliphatic chain length were selected as key structural parameters for establishing the coal macromolecular model. Following the approaches reported in Refs. [9,13,14,15], the relevant structural parameters of RNM were quantitatively evaluated from the fitted peak areas of characteristic FTIR bands.

(1) The aliphatic chain branching index is an important structural parameter for characterizing the side-chain structure of coal. In this study, it was evaluated using the relative abundance of methyl and methylene groups, expressed as the A(CH_2_)/A(CH_3_) ratio. A lower value of this parameter indicates a relatively longer aliphatic chain within the coal macromolecular structure. Based on the fitted FTIR peak areas, the aliphatic chain branching index of the RNM sample was calculated to be 0.33, suggesting that the aliphatic side chains in RNM were relatively developed.

$$\frac{A_{1}\left(CH_{2}\right)}{A_{1}\left(CH_{3}\right)}=\frac{A_{1}\left(2900\sim 2940\;cm^{-1}\right)}{A_{1}\left(2940\sim 3000\;cm^{-1}\right)}=0.33$$

(2) Aromaticity of the coal sample (I)
    Aromaticity was used to characterize the relative enrichment of aromatic functional groups with respect to the aliphatic functional groups in the coal structure. This parameter was calculated from the fitted peak areas of the characteristic aromatic and aliphatic FTIR bands. A higher aromaticity value indicates a greater contribution of aromatic structural units to the coal macromolecular framework.
  $$I=\frac{A_{1}\left(900\sim 700\;cm^{-1}\right)}{A_{1}\left(3000\sim 2800\;cm^{-1}\right)}=0.77$$

(3) Degree of aromatic ring condensation (DOC)
    DOC was employed to evaluate the condensation level of aromatic structures in the coal sample. It was defined as the ratio of the out-of-plane bending vibration area of aromatic C–H bonds in the 900–700 cm^−1^ region to the aromatic C=C skeletal vibration area near 1600 cm^−1^. This parameter reflects the relative development of condensed aromatic ring systems within the coal macromolecular structure.
  $$DOC=\frac{A_{1}\left(900\sim 700\;cm^{-1}\right)}{A_{1}\left(1600\;cm^{-1}\right)}=0.35$$

(4) Infrared aromatic carbon ratio
    The infrared aromatic carbon ratio represents the proportion of carbon atoms associated with aromatic structures relative to the total carbon atoms in coal based on the FTIR-derived aromatic and aliphatic structural parameters. In the calculation, Hal/Cal denotes the hydrogen-to-carbon atomic ratio of the aliphatic components, which was taken as 1.8 according to the literature. This parameter provides a quantitative basis for constraining the aromatic carbon content during construction of the RNM macromolecular model.
  $$f_{ar}^{C}=1-\frac{\left(\frac{A_{1}\left(2800\sim 3000\right)cm^{-1}}{A_{1}\left(700\sim 900\right)cm^{-1}\;+\;A_{1}\left(2800\sim 3000\right)cm^{-1}}\times \frac{H}{C}\right)}{\frac{H_{\mathrm{al}}}{C_{\mathrm{al}}}}=0.754$$

### 2.3. Occurrence of N and S in Coal

**Figure 2:**
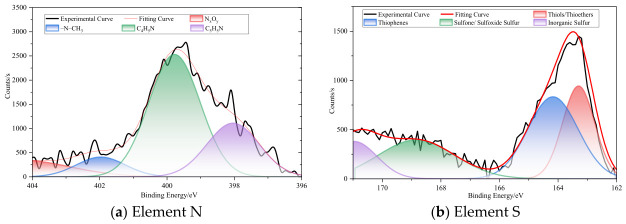
Occurrence of N and S in coal.

XPS is an effective surface analysis technique for characterizing organic molecular structures and identifying the chemical states and relative abundances of carbon-, oxygen-, nitrogen-, and sulfur-containing species in coal. Accordingly, it has been widely applied in coal structural characterization. In this study, the analysis was focused on nitrogen and sulfur species in the RNM coal sample to determine their occurrence forms and relative contents. Peak deconvolution of the N 1s and S 2p spectra was performed using Thermo Scientific Avantage V6.5 software, and the corresponding fitting results are presented in Figure 2. Figure 2 shows the Occurrence of N and S in coal.

The XPS fitting results indicated that pyrrolic nitrogen and pyridinic nitrogen were the predominant nitrogen species in the coal sample, together accounting for approximately 70–80% of the total nitrogen content. This distribution was attributed to the incorporation of pyrrolic and pyridinic nitrogen into aromatic conjugated systems, which endows these species with relatively high structural stability and enables their preservation during coalification. In contrast, oxidized nitrogen showed a relatively low abundance and was mainly derived from the oxidation of pyrrolic and pyridinic nitrogen; accordingly, the relative contents of oxidized nitrogen and quaternary nitrogen remained comparatively limited.

The S 2p fitting results showed that sulfur was mainly present in the form of thiophenic sulfur, whereas sulfone- and sulfoxide-type sulfur occurred as secondary species. With increasing coalification degree, the contents of thiol- and thioether-type sulfur generally decreased, while the proportion of thiophenic sulfur increased. This trend was ascribed to the relatively poor structural stability of sulfur-containing functional groups such as thioethers, thioesters, and thiols, which tend to transform into more stable structures under thermal maturation. Owing to its aromatic structural characteristics, thiophenic sulfur represents an important product of the transformation of unstable side-chain sulfur species and gradually becomes the dominant form of organic sulfur in the coal structure.

### 2.4. Carbon Skeleton Characteristics

**Figure 3:**
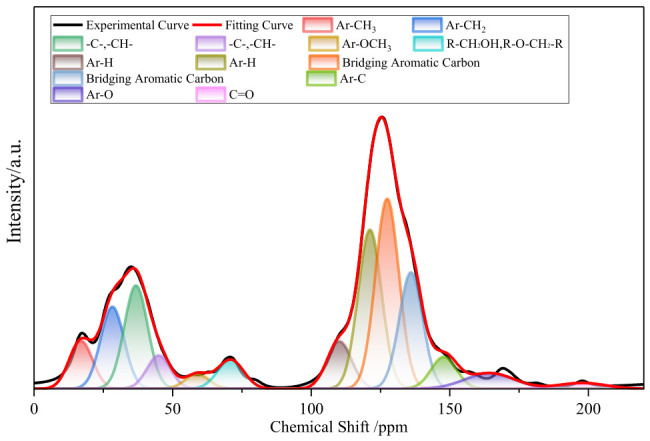
Peak fitting spectra of ^13^C NMR for coal sample.

Carbon atoms in coal macromolecules are generally classified into aliphatic and aromatic carbon species. Differences in carbon type, attached functional groups, and linkage modes give rise to distinct chemical shifts in the ^13^C NMR spectra [18]. In this study, solid-state ^13^C NMR spectroscopy was employed to qualitatively and quantitatively characterize the macromolecular structure of RNM. This technique provided direct information on the carbon skeleton and enabled further analysis of the structural features of the coal macromolecule. Figure 3 shows the Peak fitting spectra of ^13^C NMR for coal sample.

The ^13^C NMR spectrum of the weakly caking coal sample was deconvoluted using Origin software, and the fitting result showed a high degree of reliability, with an R^2^ value of 0.997. Based on the fitted peak positions and corresponding area distributions, the relative contributions of different carbon skeleton structures in RNM were quantitatively determined.

The deconvolution results indicated that the carbon skeleton of RNM was dominated by aromatic structures. The protonated aromatic carbon peaks at 110.16, 121.19, and 127.44 ppm collectively accounted for 43.89% of the total carbon signal, demonstrating that peripheral aromatic carbons were the major carbon species in the coal macromolecule. The bridging aromatic carbon signal at 136.02 ppm contributed 12.81%, while substituted aromatic carbon at 147.86 ppm accounted for 3.79%. These results suggested that the aromatic nuclei in RNM contained a certain degree of bridging and substitution, reflecting evident aromatic characteristics and a moderate condensation level.

In the aliphatic carbon region, the peaks at 16.89, 28.25, 36.79, and 44.98 ppm, assigned to aliphatic methyl, methylene, methine, and quaternary carbons, respectively, contributed a total of 29.34%. This indicated that RNM retained a certain amount of aliphatic side chains and branched structures. In particular, the relatively high proportions of methylene and methine/quaternary carbons suggested that the aliphatic moieties possessed a certain structural complexity. The methoxy and oxygen-bonded methine carbons at 58.81 and 70.92 ppm accounted for 5.20%, indicating a relatively low abundance of oxygen-containing aliphatic structures. In addition, the carboxyl/carbonyl carbon signals at 164.40 and 197.79 ppm contributed only 4.92%, further confirming the limited content of highly oxidized oxygen-containing functional groups in RNM.

Overall, the ^13^C NMR analysis demonstrated that RNM was characterized by an aromatic-dominated carbon framework, abundant peripheral aromatic carbons, moderate bridging and substitution of aromatic units, and a certain proportion of aliphatic side chains and branched structures, whereas oxygen-containing aliphatic carbons and highly oxidized carbonyl/carboxyl carbons were relatively limited.

The structural parameters used for coal molecular modeling were defined based on the solid-state ^13^C NMR analysis. *f*_al_^*^, ***f*_al_^H^**, ***f*_al_^D^**, and ***f*_al_^O^** represent the fractions of methyl carbon, aliphatic methylene/methine carbon, aliphatic methine or quaternary carbon, and oxygen-substituted aliphatic carbon, respectively. ***f*_a_^H^** denotes protonated aromatic carbon, whereas ***f*_a_^B^** represents the aromatic bridgehead carbon in condensed aromatic structures. ***f*_a__r_^C^** refers to substituted or branched aromatic carbon, generally associated with aromatic carbon bonded to alkyl or other carbon-containing substituents. ***f*_a_^P^** represents oxygen-substituted aromatic carbon, such as phenolic or phenoxy carbon, and ***f*_a_^N^** denotes non-protonated aromatic carbon, including bridgehead, substituted, and oxygenated aromatic carbons. ***f*_a_^C^** is assigned to carboxyl, carbonyl, or amide carbon. The total aliphatic carbon fraction is expressed as ***f*_al_**, while *f*_a_ represents the overall aromaticity or total aromatic carbon fraction. In addition, *f_a_^′^* denotes the aromatic ring carbon fraction, excluding carboxyl and carbonyl carbons. These parameters provide quantitative constraints for constructing representative molecular models of coal by describing the relative abundance of different carbon functional groups and aromatic structural units. Overall, the ^13^C NMR spectra can be partitioned into two major clusters: aliphatic carbon (***f*_al_** = *f*_al_^*^ + ***f*_al_^H^** + ***f*_al_^O^**) and aromatic carbon (***f*_a_^′^** = ***f*_a_^H^** + ***f*_a_^N^** = ***f*_a_^H^** + ***f*_a_^P^** + ***f*_a_^S^** + ***f*_a_^B^**). A chemical shift of 70 ppm is used as the boundary between these clusters. The structural parameters derived from the ^13^C NMR of the coal samples are summarized in Table 2.

The ratio of bridge carbons to peripheral carbons $\left(X_{BP}\right)$ was used to evaluate the degree of aromatic condensation in the coal macromolecular structure. The corresponding equations are given as follows:

(1) Ratio of Bridge Carbons to Peripheral Carbons

$$X_{BP}=\frac{f_{a}^{B}}{f_{a}^{H}+f_{a}^{P}+f_{a}^{S}}=0.25$$

(2) Average Number of Methylene Chain Carbons

$$C_{n}=\frac{f_{al}^{H}}{f_{ar}^{C}}=2.37$$

(3) Alkyl Chain Branching Index

$$BI=\frac{f_{al}^{H}}{f_{al}}\times 100\%=26.14\%$$

The calculated XBP value was 0.25, indicating that bridge carbons accounted for a relatively low proportion of the aromatic carbon framework and that the overall condensation degree of the aromatic nuclei was limited. Structurally, RNM was therefore dominated by aromatic units containing abundant peripheral carbons, whereas large, highly condensed fused-ring aromatic systems were not well developed. The $C_{n}$ value of 2.37 suggested that the aliphatic side chains in the coal sample were generally short, with methylene segments mainly consisting of approximately two to three carbon atoms, indicating limited development of long-chain aliphatic structures. In addition, the BI value of 27.13% showed that although branching existed in the aliphatic side chains, the overall branching degree remained relatively low. Thus, the aliphatic moieties in RNM were primarily characterized by relatively short and simple linear-chain structures.

### 2.5. Construction of Coal Macromolecular Models

To determine the molecular formula of the RNM macromolecule, the atomic ratios of H/C, O/C, N/C, and S/C were calculated based on the elemental analysis results. Accordingly, the empirical macromolecular formula of the coal sample was expressed as C_x_H_0.822098x_O_0.114043x_N_0.010783x_S_0.010666x_. Previous studies have suggested that the relative molecular mass of coal macromolecular structural units generally ranges from 2000 to 3000 [3,4,19]. Therefore, the molecular mass of the RNM structural unit was constrained by the following equations:

12x + 0.822098x + 16 × 0.114043x + 14 × 0.010783x + 32 × 0.010666x > 2000(1)

12x + 0.822098x + 16 × 0.114043x + 14 × 0.010783x + 32 × 0.010666x < 3000(2)

Meanwhile, the number of atoms for each element was required to be a positive integer. On the basis of the above constraints, together with the aromatic carbon fraction (f_a_) and aliphatic carbon fraction (***f*_al_**) obtained previously, the number of aromatic and aliphatic carbons was calculated to be 61 and 115, respectively. Consequently, the molecular formula of the RNM structural unit was determined as C_176_H_156_N_2_O_19_S_2_.

To further clarify the aromatic structural composition of the RNM macromolecule, the types and quantities of aromatic structural units were determined using the carbon skeleton parameter $X_{BP},\;$derived from the ^13^C NMR analysis, in combination with the total aromatic carbon content. The $X_{BP}$ values assigned to benzene, naphthalene, anthracene/phenanthrene, and pyrene rings were 0, 0.25, 0.40, and 0.50, respectively, whereas those of pyrrole, pyridine, and thiophene were all taken as 0. The calculated $X_{BP}$ value of RNM was 0.25. Based on these parameters, the number of benzene, naphthalene, anthracene/phenanthrene, pyrene, pyridine, pyrrole, and thiophene units in the coal structural unit was determined to be 3, 3, 3, 0, 1, 1, and 2, respectively.

**Figure 4:**
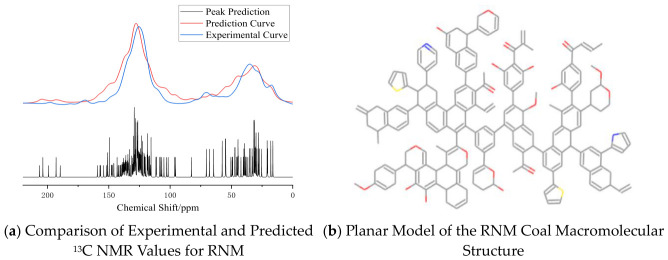
Planar model of the macromolecular structure of coal and its verification diagram.

Based on the above structural constraints, a two-dimensional macromolecular model of RNM was constructed using KingDraw software. The constructed model was subsequently imported into MestReNova software for spectral validation. By adjusting the spatial arrangement and bonding modes of different functional groups in the model, the simulated 13C NMR spectrum of the constructed macromolecular structure was compared with the experimentally obtained ^13^C NMR spectrum. The comparison showed good consistency between the simulated and experimental spectra, indicating that the established RNM macromolecular model could reasonably represent the carbon skeleton and functional group distribution of the coal sample. Planar model of the macromolecular structure of coal and its verification diagram is shown in Figure 4.

### 2.6. DFT Calculations

Before performing DFT optimization on the macromolecule, the initial coal macromolecule model was first subjected to geometric optimization, annealing optimization, and kinetic equilibrium using the Forcite module in Materials Studio software 2023 to achieve basic molecular dynamic structural stability. This operation was also to prepare for DFT optimization. If DFT optimization is performed directly, the structural and energy changes may be too large, resulting in excessively long calculation time or even error problems. The simulation parameters are as follows: For geometry optimization, the COMPASS II force field is used with fine computational accuracy. The charges term is set to force field-assigned. Van der Waals and electrostatic interactions can be performed using an atom-based method with a cutoff radius of 12.5 Å. For Annel optimization, an NVT ensemble is used. The temperature range is set to 300–600 K. The annealing cycles are set to five cycles. The convergence criteria are set as follows: energy change threshold of 1.0 × 10^−4^ kcal/mol, maximum force threshold of 5.0 × 10^−3^ kcal/mol/Å, and maximum displacement threshold of 5.0 × 10^−5^ Å. The number of kinetic steps for each temperature stage can be set to 2000 steps. Temperature control was performed using the Nose thermostat. Kinetic optimization was performed using the NVT ensemble, with the temperature set to 298 K, the time step to 1.0 fs, and the total simulation time to 500 ps. The force field, charges, van der Waals and electrostatic interactions, and cutoff radius were selected in the same way as in geometry optimization.

DFT calculations were performed on the optimized RNM molecular model to identify the charge-distribution features and potential reaction sites. Electrostatic potential (ESP), frontier molecular orbitals, and Fukui functions were analyzed to evaluate the spatial selectivity of radical, electrophilic, and nucleophilic attacks [20]. These descriptors were used to determine the regions most likely to participate in oxygen adsorption and subsequent oxidation reactions.

The optimization was conducted within the framework of DFT using a geometry optimization task. The coal macromolecule was considered as an isolated aperiodic molecular system with zero total charge. The electron exchange correlation function adopts the PBE functional based on the GGA, uses the Grimme method for DFT-D weak interaction correction, uses DFT Semi-core Pseudopods, and selects the dual numerical polarization basis set DNP for kernel processing, and sets the accuracy to fine. The electron spin is not restricted and symmetry is used. The SCF convergence criterion was set to 1.0 × 10^−6^ Ha, and the maximum SCF cycle count was set to 200. The convergence criteria for geometry optimization were set as follows: energy change 1.0 × 10^−5^ Ha, maximum force 2.0 × 10^−3^ Ha/Å, maximum displacement 5.0 × 10^−3^ Å, and maximum optimization steps 500. During the optimization process, all atoms were fully relaxed, allowing the aromatic clusters, aliphatic side chains, and oxygen-containing functional groups in the coal macromolecules to reach stable configurations. Subsequently, the optimized structure was used for electronic structure and reactivity analysis.

**Figure 5:**
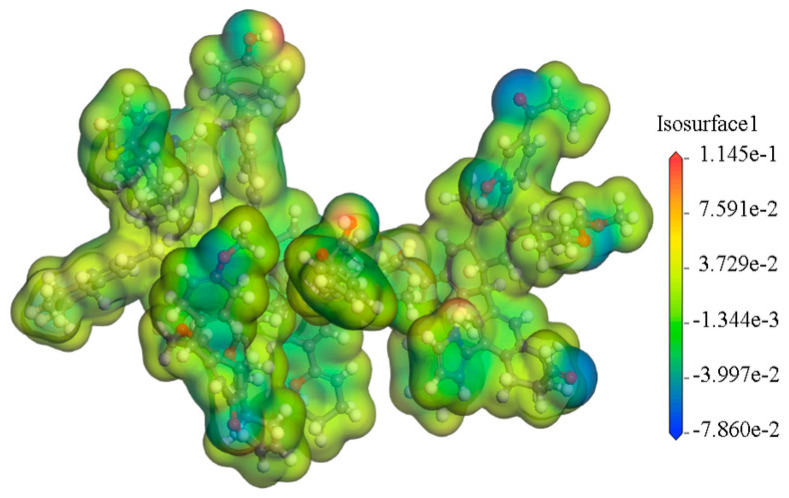
Electrostatic potential distribution of RNM.

The electrostatic potential distribution of RNM is shown in Figure 5. The ESP surface was dominated by nearly neutral regions; however, pronounced local polarization was observed around heteroatom-containing functional groups and their adjacent structural units. Negative ESP regions, shown in blue, were mainly localized near oxygen- and nitrogen-containing sites, whereas positive ESP regions, shown in red, were concentrated around hydroxyl hydrogen atoms and selected peripheral positions. This distribution indicated that the molecular polarity of RNM was primarily governed by peripheral heteroatom-containing functional groups and neighboring moieties. Owing to the pronounced charge density fluctuation in these regions, they could provide diverse active sites for the oriented adsorption of polar molecules, hydrogen bond formation, and initial chemical reactions.

**Figure 6:**
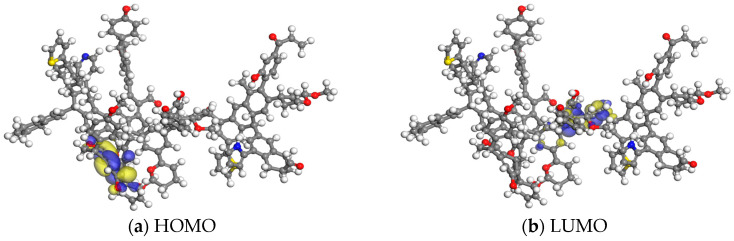
Frontier orbital distribution of RNM.

The frontier molecular orbital distribution of RNM is shown in Figure 6. HOMO was mainly localized in the fused-ring edge regions enriched with N and S heteroatoms and around localized oxygen-containing structures in the central region. This distribution indicated that these regions possessed relatively high electron density and strong electron-donating ability, and therefore were more likely to participate preferentially in electrophilic reactions [21]. In contrast, LUMO was more broadly distributed over the central bridged aromatic sheets and adjacent conjugated regions, extending toward several oxygen-substituted structures. These sites were associated with highly conjugated and relatively electron-deficient skeletons. The partial spatial separation, together with local overlap between electron-donating and electron-accepting regions, could facilitate intramolecular charge redistribution and subsequent chemical reactions, thereby inducing local changes in bond order and promoting reaction activation.

**Figure 7:**
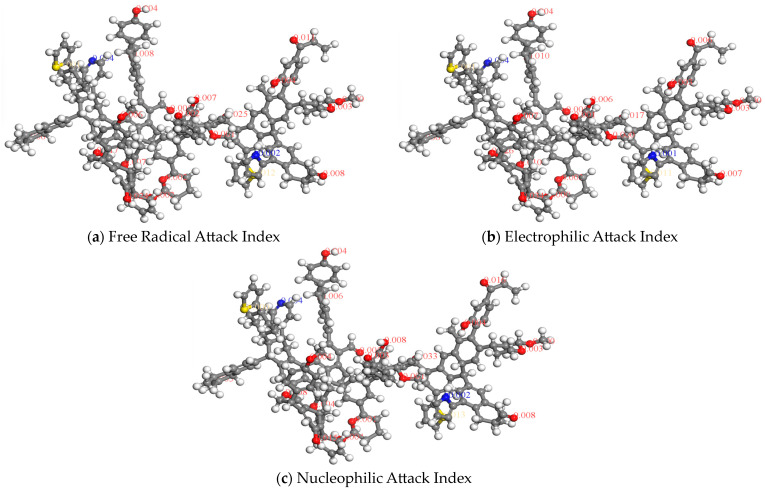
Reactivity attack indices of RNM.

The reactivity attack indices of RNM are presented in Figure 7. The radical, electrophilic, and nucleophilic attack indices calculated from the Fukui function showed that the reactive sites in the coal molecule exhibited distinct spatial selectivity. Regions with relatively high index values were mainly concentrated around oxygen-containing functional groups located at the molecular periphery and their adjacent carbon atoms. The highest local reactivity response appeared near the oxygen-containing terminal groups, with a value of approximately 0.024. The central bridging segments and their neighboring sites showed the second-highest response, with values of approximately 0.020. In addition, the terminal oxygen-containing side chains on the upper-right side and several oxygen-containing sites in the upper region also exhibited relatively high reactivity, with values of approximately 0.011 and 0.008, respectively. The nitrogen-containing sites generally showed low index values, whereas the sulfur-containing sites exhibited greater variability, indicating that their reactivity was more strongly affected by the local conjugated environment and neighboring substituents.

High radical attack indices were mainly distributed on oxygen-containing side chains, bridging sites, and adjacent carbon atoms, suggesting that these regions were more prone to localized unpaired electron formation and homolytic bond cleavage. Therefore, they could serve as preferential initiation sites for radical-induced oxidation and skeleton fragmentation. During oxidation, radical chain reactions, or interactions with polar reagents, the initial reactions were thus more likely to occur at peripheral oxygen-containing structures and active bridging sites. Overall, RNM exhibited a reactivity pattern characterized by a relatively stable aromatic core and highly activated peripheral heteroatom-containing functional groups.

### 2.7. Adsorption Differences and Competitive Adsorption Mechanisms of O_2_ and CO_2_

To establish a coal matrix adsorption model, the Construction task of Amorphous Cell was used, with optimized coal macromolecules as the input component. The number of molecules was set to 10, the cell type to periodic cubic cell, the space group to P1, and the initial density to 0.5 g/cm^3^ to avoid severe atomic overlap caused by direct high-density packing. Density equilibrium was achieved using an NPT ensemble, with the temperature set at 298.15 K and the initial compaction pressure set at 0.1 GPa. The simulation time was 200 ps. The pressure was then set to 0.5 GPa for compaction, and the simulation time was 200 ps. Temperature control was achieved using a Nose thermostat, and pressure control using a Berendsen barostat. The convergence criteria for geometric optimization were the same as above. The final compacted density was 1.18 g/cm^3^. After structural optimization and molecular dynamics equilibration, stable and reasonable cell parameters were obtained. The lattice type was 3D triclinic, with cell dimensions of OA = OB = OC = 44.577664 Å.

The adsorption heats reported in this study were obtained from force field-based grand canonical Monte Carlo simulations. The COMPASS III force field was employed to describe the interactions among the coal matrix and gas molecules. Nonbonded interactions were treated by considering both van der Waals and electrostatic contributions.

**Figure 8:**
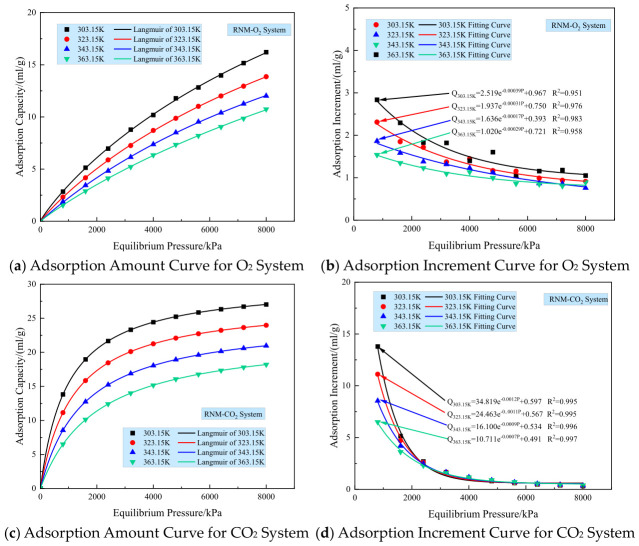
Adsorption kinetics curves of O_2_ and CO_2_ on RNM at different temperatures.

To elucidate the microscopic adsorption differences between O_2_ and CO_2_ in the coal macromolecular structure at different temperatures, isothermal adsorption simulations were performed using the Sorption module in Materials Studio 2023. The geometrically and dynamically optimized RNM molecular model was used as the adsorption substrate, and adsorption equilibrium calculations were conducted using the grand canonical Monte Carlo method at 303.15, 323.15, 343.15, and 363.15 K. To ensure the comparability of the simulation results among different temperatures and gas systems, all calculations were performed using the COMPASS III force field. The atomic charges were assigned using the “force field assigned” method, and the cutoff radius was set to 15.5 Å [22]. Figure 8a–d show the adsorption isotherms and incremental adsorption curves of O_2_ and CO_2_ in the RNM model over the pressure range of 0–8 MPa. The Langmuir fitting parameters obtained from the adsorption data are summarized in Table 3. The gas adsorption capacity calculated in the Sorption module is expressed in units of “molecules per unit cell”—specifically, the number of adsorbate molecules present within the current unit cell—and is subsequently converted into the commonly used laboratory units of mmol/g and mL/g in accordance with Equation (3).

(3) $$uptake/(mL/g)=22,400\times \frac{N}{M_{cell}}$$

In this equation, $M_{cell}$ is the relative molecular mass of the coal molecule (g/mol), and N is the number of adsorbed molecules.

As shown in Figure 8, the adsorption capacities of both O_2_ and CO_2_ in RNM increased with increasing equilibrium pressure within the temperature range of 303.15–363.15 K, exhibiting typical Langmuir-type adsorption behavior. In the low-pressure region, gas molecules rapidly occupied the high-energy adsorption sites in the coal molecular model, resulting in a sharp increase in adsorption capacity. With further increases in pressure, the number of available adsorption sites gradually decreased, and the adsorption curves progressively approached a plateau. At the same equilibrium pressure, the adsorption capacity of O_2_ was markedly lower than that of CO_2_, indicating a stronger adsorption affinity of RNM toward CO_2_.

Temperature exerted a significant influence on the adsorption behavior of both gases. As the temperature increased from 303.15 to 363.15 K, the adsorption capacities of O_2_ and CO_2_ both decreased, suggesting that elevated temperature enhanced the thermal motion of gas molecules and weakened their stable adsorption on the pore surfaces and active sites of the coal structure. At approximately 8000 kPa, the O_2_ adsorption capacity decreased from about 16.2 to 10.7 mL/g, corresponding to a reduction of approximately 34%. Under the same pressure condition, the CO_2_ adsorption capacity decreased from about 27.0 to 18.2 mL/g, with a reduction of approximately 33%. These results indicated that the adsorption of O_2_ and CO_2_ in RNM was thermally unfavorable at elevated temperature and was dominated by physical adsorption interactions.

The adsorption parameters in Table 4 further confirm the temperature-dependent adsorption behavior of O_2_ and CO_2_ in RNM. For the single-component O_2_ system, the adsorption capacity decreased from 0.064 to 0.038 mL/g as the temperature increased from 303.15 to 363.15 K, corresponding to a reduction of approximately 40.6%. Meanwhile, the adsorption heat of O_2_ decreased from 2.681 to 2.550 kcal/mol, indicating relatively weak interactions between O_2_ and the coal matrix and suggesting that O_2_ adsorption was mainly governed by physical adsorption. In the O_2_-CO_2_ competitive system, the total adsorption capacity decreased from 0.612 to 0.242 mL/g with increasing temperature. CO_2_ adsorption decreased from 0.571 to 0.218 mL/g but remained dominant throughout the temperature range, whereas O_2_ adsorption remained only within 0.041–0.024 mL/g. The proportion of adsorbed O_2_ increased slightly from 6.7% to 9%, implying that CO_2_ adsorption was more sensitive to temperature. Compared with the single-component O_2_ system, O_2_ adsorption in the competitive system decreased by approximately 36%, demonstrating the competitive inhibitory effect of CO_2_. In addition, the adsorption heat of CO_2_ was 5.667–4.796 kcal/mol, nearly twice that of O_2_, indicating stronger interactions between CO_2_ and the oxygen-containing functional groups, aromatic structures, and pore surfaces of RNM.

The adsorption difference between O_2_ and CO_2_ was more evident in the low-pressure region. At approximately 2500 kPa, the CO_2_ adsorption capacities were about 21.5, 18.5, 15.3, and 12.5 mL/g at 303.15, 323.15, 343.15, and 363.15 K, respectively, whereas the corresponding O_2_ adsorption capacities were only 6.8, 5.8, 4.9, and 4.2 mL/g. This indicated that CO_2_ preferentially interacted with the pore surfaces, aromatic structures, and oxygen-containing functional groups of RNM. The incremental adsorption curves showed that both gases gradually approached adsorption saturation at high pressure, while CO_2_ exhibited a higher adsorption increment in the low-pressure region and occupied high-energy adsorption sites more rapidly [18]. The Langmuir parameter b decreased from 1.525 to 0.658 for O_2_ and from 1.052 to 0.501 for CO_2_ with increasing temperature, further confirming that elevated temperature weakened gas–coal interactions.

**Figure 9:**
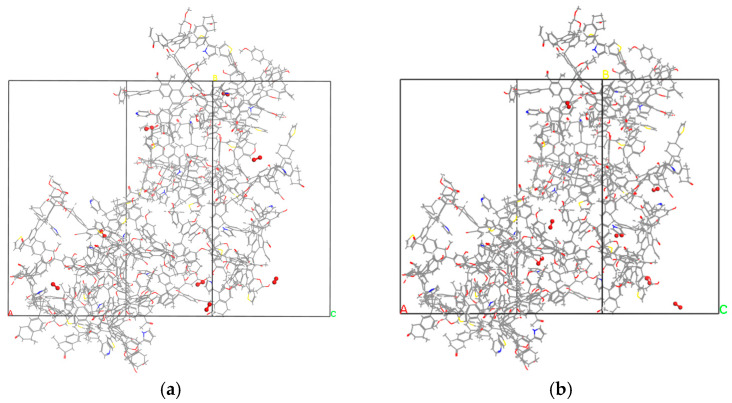

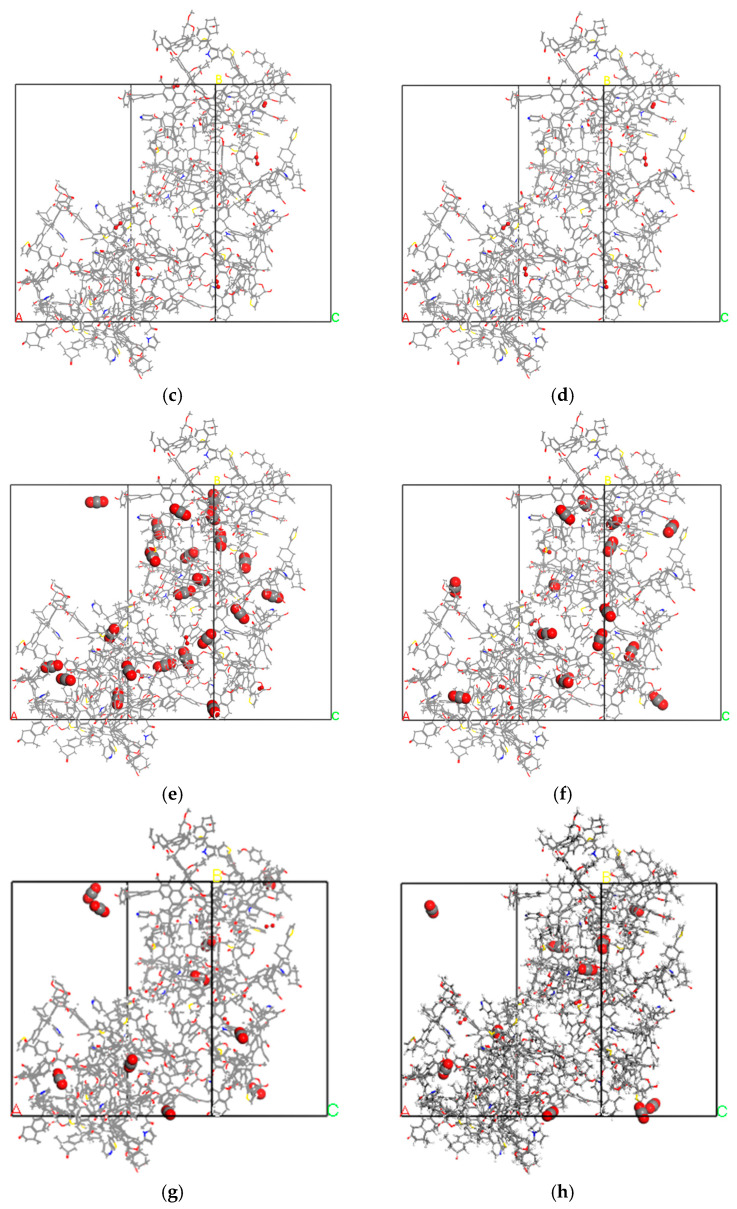
Competitive adsorption configurations of O_2_ and O_2_-CO_2_ at 303.15–363.15 K. (**a**) Adsorption configurations of O_2_ on RNM at 303.15 K. (**b**) Adsorption configurations of O_2_ on RNM at 323.15 K. (**c**) Adsorption configurations of O_2_ on RNM at 343.15 K. (**d**) Adsorption configurations of O_2_ on RNM at 363.15 K. (**e**) The competitive adsorption configuration of O_2_–CO_2_ on RNM at 303.15 K. (**f**) The competitive adsorption configuration of O_2_–CO_2_ on RNM at 323.15 K. (**g**) The competitive adsorption configuration of O_2_–CO_2_ on RNM at 343.15 K. (**h**) The competitive adsorption configuration of O_2_–CO_2_ on RNM at 363.15 K.

Figure 9 shows the competitive adsorption configurations of O_2_ and O_2_-CO_2_ at 303.15–363.15 K. To clearly illustrate the adsorption effects of RNM on O_2_ and CO_2_, the RNM coal matrix is presented in a line model, O_2_ molecules are shown as ball-and-stick models, and CO_2_ molecules are shown in CPK form in Figure 9a–h. The adsorption configurations in Figure 9 provided molecular-level evidence for the above results. With increasing temperature, the number and local enrichment of O_2_ molecules decreased, indicating reduced adsorption stability. In the O_2_-CO_2_ competitive system under equal partial pressures, CO_2_ molecules showed significantly higher spatial occupation than O_2_ at all temperatures. The presence of CO_2_ compressed the available adsorption space for O_2_ and reduced its adsorption amount, confirming that CO_2_ preferentially occupied effective adsorption sites in RNM. This behavior was mainly attributed to the higher polarizability of CO_2_ and its stronger van der Waals and electrostatic interactions with oxygen- and nitrogen-containing functional groups and aromatic structures [10].

Overall, CO_2_ was more readily adsorbed by the RNM macromolecular structure than O_2_, while increasing temperature weakened the adsorption stability of both gases. The adsorption isotherms, adsorption heat data, and molecular configurations consistently demonstrated that CO_2_ inhibited O_2_ adsorption through competitive site occupation, reduced O_2_ enrichment, and limited O_2_ access to reactive sites [12,13]. These findings provide a microscopic basis for understanding O_2_-CO_2_ competitive adsorption, CO_2_-assisted oxygen inhibition, and gas migration during coal–oxygen complexation.

The adsorption configurations of single-component O_2_ and O_2_-CO_2_ competitive adsorption in the RNM system are shown in Figure 9. With increasing temperature, both the residence number and local enrichment degree of the O_2_ molecules gradually decreased, and the adsorption sites became more dispersed. This result indicated that elevated temperature enhanced the thermal motion of O_2_ molecules, thereby weakening the van der Waals interactions and local electrostatic interactions between O_2_ and the coal molecular skeleton. Consequently, the adsorption stability of O_2_ in the coal pore structure decreased. This phenomenon was consistent with the trend observed in the adsorption isotherms, in which the O_2_ adsorption capacity decreased with increasing temperature [13,23,24,25].

In the O_2_-CO_2_ competitive adsorption system, where the two gases were introduced under equal partial-pressure conditions, the adsorption amount and spatial occupation of CO_2_ molecules in the RNM model were significantly greater than those of O_2_. Owing to the relatively weak interaction between O_2_ molecules and the coal matrix, the coexistence of CO_2_ further reduced the available adsorption sites for O_2_, resulting in a lower O_2_ adsorption amount in the competitive system than in the single-component O_2_ system. This demonstrated that CO_2_ preferentially occupied the effective adsorption sites in the coal structure, exhibited a stronger adsorption advantage at all investigated temperatures, and exerted an inhibitory effect on O_2_ adsorption [12]. This behavior was mainly attributed to the stronger polarizability of CO_2_ molecules and their stronger van der Waals and electrostatic interactions with oxygen-containing and nitrogen-containing functional groups as well as aromatic structures in coal. The configuration analysis indicated that RNM possessed a higher adsorption affinity for CO_2_ than for O_2_, while increasing temperature reduced the adsorption stability of both gases in the coal structure.

Overall, the adsorption affinity of RNM for CO_2_ was significantly stronger than that for O_2_. Although elevated temperature weakened the adsorption stability of both gases in the RNM model, it did not alter the overall tendency of preferential CO_2_ adsorption. The competitive inhibitory effect of CO_2_ on O_2_ was further verified at the molecular configuration level, providing a theoretical basis for understanding O_2_/CO_2_ competitive adsorption in coal, oxygen inhibition by CO_2_ injection, and gas migration and site occupation behavior during coal–oxygen complexation.

## 3. Discussion

This study establishes a molecular-level framework for interpreting CO_2_ inhibition of coal–oxygen complexation in weakly caking coal by integrating structural characterization, DFT reactivity analysis, and GCMC adsorption simulation. The constructed RNM macromolecular model indicates a moderately aromatic but weakly condensed carbon framework. Meanwhile, abundant oxygen-containing functional groups and aliphatic side chains provide structural sites for gas adsorption and low-temperature oxidation. This structural feature explains why RNM possesses both relatively stable aromatic domains and highly reactive peripheral functional groups.

The DFT results further reveal the intrinsic origin of coal–oxygen reactivity. Electrostatic potential, frontier orbital, and Fukui function analyses consistently indicate that the active regions are mainly located around peripheral oxygen-containing groups, bridge-linked segments, and adjacent carbon atoms. The Fukui index calculations show that coal–oxygen complexation is not uniformly distributed across the coal molecule, but is rather preferentially initiated at heteroatom-containing edge structures and bridge-linked active sites.

The adsorption simulation results clarify the competitive inhibition mechanism of CO_2_. Both O_2_ and CO_2_ adsorption conform to the Langmuir model, but RNM exhibits a much stronger affinity for CO_2_ than for O_2_. The results demonstrate that CO_2_ suppresses coal–oxygen complexation mainly through preferential adsorption, competitive site occupation, reduction in local O_2_ enrichment, and obstruction of O_2_ access to oxidation-active sites.

The main innovation of this work lies in linking the “reactive site distribution” obtained from DFT calculations with the “competitive adsorption behavior” obtained from GCMC simulations. Rather than only comparing adsorption capacities, this study identifies where O_2_ is likely to react and how CO_2_ prevents O_2_ from reaching these sites. This provides a molecular explanation for CO_2_-assisted oxygen inhibition in weakly caking coal. The theoretical significance is that it deepens the understanding of gas adsorption, migration, and site occupation during coal low-temperature oxidation and provides a microscopic basis for CO_2_ injection, oxygen suppression, and prevention of coal spontaneous combustion.

## 4. Materials and Methods

### 4.1. Elemental Analysis and Proximate Analysis

The elemental composition of the coal sample was determined using an Elementar Unicube elemental analyzer (Elementar, Langenselbold, Germany). Proximate analysis was performed to determine the moisture content, ash yield, volatile matter, and fixed carbon.

For moisture determination, approximately 1.0 g of a coal sample was placed in a pre-dried crucible and dried at 110 °C for 1 h until a constant mass was obtained. The sample was then cooled in a desiccator and weighed to calculate the moisture content. For ash determination, the dried coal sample was heated in a muffle furnace, with the temperature gradually increased to 800 °C and maintained until the sample mass became constant. Volatile matter was determined by placing approximately 1.0 g of coal sample in a covered crucible and heating it at 900 °C for 7 min in a high-temperature furnace. The fixed carbon content was calculated by the difference. All measurements were conducted in duplicate to ensure the reliability of the analytical results.

### 4.2. FTIR Experiment

FTIR was performed using a Nicolet iS 20 infrared spectrometer (Thermo Fisher Scientific, Waltham, MA, USA). The KBr pellet method was adopted for spectral acquisition. Briefly, approximately 1.0 mg of finely ground coal powder was mixed with approximately 100 mg of spectroscopic-grade KBr and thoroughly ground to ensure homogeneous dispersion. The mixture was subsequently compressed into a transparent pellet under a pressure of 15 MPa using a hydraulic press.

Before sample measurement, a background spectrum was collected using a blank KBr pellet. The FTIR spectra of the coal samples were recorded over the wavenumber range of 4000–400 cm^−1^, with a spectral resolution of 4 cm^−1^ and 64 scans. Baseline correction, background subtraction, and normalization were then performed to improve spectral comparability and facilitate the identification of functional groups. The obtained spectra were further processed and analyzed using Origin 2024b software.

### 4.3. XPS Experiment

XPS was conducted using a K-Alpha X-ray photoelectron spectrometer (Thermo Fisher Scientific, Waltham, MA, USA) to characterize the chemical states of heteroatoms in the coal sample. Prior to analysis, the powdered coal sample was either pressed into pellets or uniformly dispersed onto indium foil sample holders and then introduced into an ultra-high-vacuum chamber with a pressure of approximately 10^−9^ mbar.

A monochromatic Al Kα X-ray source with a photon energy of 1486.6 eV was used for excitation. Survey spectra were first collected to determine the surface elemental composition of the coal sample. Subsequently, high-resolution spectra of selected regions, including N 1s and S 2p, were acquired for detailed chemical state analysis. The binding energies were calibrated using the C 1s peak at 284.8 eV as the reference. The acquired spectra were processed via background subtraction and peak deconvolution to identify the occurrence forms of nitrogen- and sulfur-containing species in the coal sample. Data baseline and peak fitting were performed using Avantage 6.6.0 software.

### 4.4. ^13^C NMR Experiment

The carbon structural characteristics of the coal sample were analyzed using ^13^C NMR spectroscopy on a Bruker Avance Neo 400 WB spectrometer (Bruker, Bremen, Germany). The cross-polarization magic-angle spinning technique was employed for spectral acquisition. Approximately 100 mg of finely ground coal powder was packed into a 4 mm zirconia rotor and sealed tightly prior to measurement.

During the experiment, the rotor spinning speed was maintained at 8–12 kHz to minimize spinning sidebands. The contact time was set to 1–2 ms, and the recycle delay was set within the range of 1–5 s according to the relaxation behavior of the coal sample. Tetramethylsilane was set to 0 ppm, and the carboxyl carbon signal at δ = 176.5 ppm was used as an external standard, serving as the basis for automatic baseline correction. The obtained spectra were further processed and analyzed using Origin 2024b software.

### 4.5. Quantum Mechanical Calculations of Coal Model

Quantum chemical calculations were performed on the optimized RNM macromolecular model using Materials Studio 2023 software. The initial structure was first geometrically optimized using the Forcite module to obtain a stable, energy-minimized configuration. Subsequently, density functional theory calculations were conducted using the DMol^3^ module to evaluate the electrostatic potential distribution, frontier molecular orbitals, and reactive attack indices of the coal molecule. The electrostatic potential map was used to identify charge-enriched and charge-deficient regions, thereby revealing potential sites for polar adsorption and chemical interaction. The spatial distributions of the highest occupied molecular orbital and lowest unoccupied molecular orbital were analyzed to determine the electron-donating and electron-accepting regions of the molecule, respectively. Furthermore, the radical, electrophilic, and nucleophilic attack indices were calculated based on the Fukui function to quantitatively characterize the reactivity of different atomic sites. These calculations were used to clarify the spatial distribution of active sites in RNM and to evaluate their potential roles in coal–oxygen complexation reactions.

## 5. Conclusions

(1) The molecular formula of RNM was determined as C_176_H_156_N_2_O_19_S_2_. Its molecular structure was dominated by aromatic carbon, with an aromaticity of 65.34%. The bridge-carbon-to-peripheral-carbon ratio was 0.25, indicating that the aromatic structure exhibited a certain degree of condensation, although the overall condensation degree remained limited. In addition, the coal model contained abundant oxygen-containing functional groups and aliphatic side chains, which provided potential active sites for gas adsorption and low-temperature oxidation reactions.
(2) DFT calculations showed that the high-electrostatic-potential regions and high-Fukui-index regions of RNM were mainly distributed around peripheral oxygen-containing functional groups, bridging chain segments, and their adjacent carbon atoms. The maximum reactivity index was approximately 0.024, indicating that these regions were more susceptible to radical attack and oxidation reactions. Therefore, they could serve as preferential activation sites during coal–oxygen complexation.
(3) The adsorption behaviors of both O_2_ and CO_2_ in RNM conformed to the Langmuir equation. RNM exhibited a significantly stronger adsorption affinity for CO_2_ than for O_2_, and CO_2_ adsorption tended to approach saturation at approximately 4000 kPa. In the single-component adsorption system, the CO_2_ adsorption capacity at 8000 kPa was approximately 1.6 times that of O_2_. In the binary competitive adsorption system, the CO_2_ adsorption capacity ranged from 0.571 to 0.218 mL/g, whereas that of O_2_ was only 0.041–0.024 mL/g, indicating that CO_2_ preferentially occupied pore spaces and high-energy adsorption sites in RNM. Although the O_2_ adsorption capacity decreased with increasing temperature, its relative proportion increased from 6.70% to 9.00%, suggesting that elevated temperature had a more pronounced destabilizing effect on CO_2_ adsorption. These results indicate that the inhibitory effect of CO_2_ on coal–oxygen complexation was mainly manifested through competitive adsorption site occupation, reduced O_2_ enrichment, and decreased probability of oxidation reactions at active sites.

## Figures and Tables

**Table 1:** Elemental analysis and proximate analysis of coal sample.

Sample	Proximate Analysis/%				Elemental Analysis/%				
	M_ad_	A_ad_	V_ad_	FC_ad_	C	H	O	N	S
RNM	0.88	6.67	38.92	53.53	73.13	5.01	11.12	0.92	2.08

**Table 2:** Structural parameters derived from ^13^C NMR of coal sample.

Sample	*f*_al_^*^	*f*_al_^H^	*f*_al_^O^	*f*_a_^H^	*f*_a_^B^	*f*_a__r_^C^	*f*_a_^P^	*f*_a_^N^	*f*_a_^C^	*f*_al_	*f*_a_	*f*_a_^′^
RNM	5.27	9.03	5.20	43.89	12.81	3.79	3.61	20.21	1.31	34.54	65.41	64.10

**Table 3:** Langmuir constants of O_2_ and CO_2_ on RNM at different temperatures.

Adsorbent Gas	Temperature/K	Langmuir Coefficient			R^2^
		a	b	c	
O_2_	303.15	48.267	1.525	0.985	0.9956
	323.15	39.969	1.145	0.061	0.9964
	343.15	33.875	0.769	0.012	0.9978
	363.15	41.600	0.658	0.047	0.9951
CO_2_	303.15	30.244	1.052	0.410	0.9946
	323.15	27.507	0.853	0.614	0.9982
	343.15	25.008	0.756	0.756	0.9967
	363.15	22.756	0.501	0.335	0.9974

**Table 4:** Heat of adsorption for O_2_ and CO_2_ at 20 kPa.

Temperature/K	O_2_ Adsorption Heat/ kcal/mol	CO_2_ Adsorption Heat/ kcal/mol	CO_2_/O_2_ Heat Ratio
303.15	2.681	5.667	2.11
323.15	2.637	5.374	2.04
343.15	2.594	5.083	1.96
363.15	2.550	4.796	1.88

## Data Availability

The original contributions presented in this study are included in the article. Further inquiries can be directed to the corresponding author.

## Abbreviations

The following abbreviations are used in this manuscript:

FTIR	Fourier transform infrared spectroscopy
XPS	X-ray photoelectron spectroscopy
^13^C NMR	Solid-state ^13^C nuclear magnetic resonance
RNM	Weakly caking coal from Dahaize mine
HOMO	Highest occupied molecular orbital
LUMO	Lowest unoccupied molecular orbital
DFT	Density functional theory
GCMC	Grand canonical Monte Carlo
ESP	Electrostatic potential
CBM	Coalbed methane
